# The Association Among Social Support, Self-Efficacy, Use of Mobile Apps, and Physical Activity: Structural Equation Models With Mediating Effects

**DOI:** 10.2196/12606

**Published:** 2019-09-25

**Authors:** Taotao Wang, Mengyuan Ren, Ying Shen, Xiaorou Zhu, Xing Zhang, Min Gao, Xueying Chen, Ai Zhao, Yuhui Shi, Weizhong Chai, Xinchuan Liu, Xinying Sun

**Affiliations:** 1 Department of Social Medicine and Health Education School of Public Health Peking University Beijing China; 2 Institute of Reproductive and Child Health School of Public Health Peking University Beijing China; 3 Department of Nutrition and Food Hygiene School of Public Health Peking University Beijing China; 4 School of Journalism and Communication Peking University Beijing China

**Keywords:** mobile apps, physical activity, social support, self-efficacy, structural equation modeling

## Abstract

**Background:**

Physical inactivity is a risk factor for chronic noncommunicable diseases. Insufficient physical activity has become an important public health problem worldwide. As mobile apps have rapidly developed, physical activity apps have the potential to improve the level of physical activity among populations.

**Objective:**

This study aimed to evaluate the effect of physical activity apps on levels of physical activity among college students.

**Methods:**

A Web-based questionnaire was used to survey college students in Beijing from December 27, 2017, to January 5, 2018. According to a previous survey, 43% of college students using physical activity apps and 36% of those who never used such apps achieved the physical activity recommendations. In this study, the sample size was calculated to be 500. The questionnaire consisted of 5 parts: the use of physical activity apps, sports habits, social support, self-efficacy, and social demographic information. Structural equation modeling was used to test the relationships between the use of physical activity apps, self-efficacy, social support, and level of physical activity.

**Results:**

Of the 1245 participants, 384 college students (30.8%) used physical activity apps (in the past month). Of these 384 students, 191 (49.7%) gained new friends via the app. College students who were using physical activity apps had a higher level of physical activity and higher scores for social support and self-efficacy (*P*<.001) than those who did not use such apps. The use of physical activity apps significantly affected the mediating effect of physical activity level through social support (beta=.126; *P*<.001) and self-efficacy (beta=.294; *P*<.001). Gender played an important role in app use, self-efficacy, and physical activity in the mediation model: male users spent more time on physical activity and had higher self-efficacy scores (*P*<.001).

**Conclusions:**

This study focused on college students in Beijing and found that the use of physical activity apps is associated with higher physical activity levels among these students. This effect is mainly through the mediation effect of social support and self-efficacy, rather than the direct effect of physical activity apps. The use of physical activity apps is associated with a higher social support level and higher self-efficacy score. Furthermore, a high social support level and high self-efficacy score are associated with higher physical activity levels.

## Introduction

Physical activity is an important foundation of general health. People’s way of life has changed dramatically, including changes in diet, a decrease in physical activity, and increase in tobacco use [1]. Insufficient physical activity has become an important public health problem worldwide [2] and is associated with the progression of many chronic diseases [3]. A survey on disease risk factors conducted by the World Health Organization (WHO) indicated that physical inactivity has become one of the 4 leading causes of death [4]. The International Physical Activity Questionnaire (IPAQ) classifies physical activity as occupational, domestic, traffic, and leisure activities. In terms of the metabolic equivalent of various activities, the intensity of physical activity can be delineated as high, moderate, and low [5], which are distinguished according to the change in heartbeat, physiological sensation, and energy consumption. Moderate-intensity physical activities (MPA) are those that may cause slight sweating and make the heart beat slightly faster. Vigorous-intensity physical activities (VPA) lead to excessive sweating and make the heart beat significantly. The WHO recommends that adults aged 18 to 64 years allocate at least 150 min a week for MPA, or 75 min a week for VPA, or an equivalent combination of MPA and VPA [4].

Globally, 80.3% of adolescents aged 13 to 15 years do not achieve the current physical activity recommendations [6]. In China, only 18.7% of adults aged 20 to 69 years achieve the current physical activity recommendations [7]. Furthermore, physical activity tends to decline with age throughout adolescence [8,9] and tracks into adulthood [10]. In the case of college students, who are in the transition from adolescence to adulthood, developing good physical activity habits can help them maintain a good physical health. Thus, the college age is an important time for intervention. Many factors affect physical activity levels. For example, demographic characteristics such as age, gender, and education level are associated with physical activity level, environmental factors, individual physical health, and psychological factors [11-13]. Furthermore, numerous studies have shown that social support and self-efficacy are among the most important factors affecting the physical activity level [14-16]. As these 2 factors are so important and can be improved through interventions [17], we focused on them in this study.

With the development of the internet and mobile phones, the number of mobile phone apps for improving physical activity has been increasing in recent years [18]. In China, many mobile phone apps, such as KEEP, Gu Dong, and Yue Dongquan, can help people participate in physical activity. Physical activity apps can be roughly delineated as fitness and running apps. This review showed that physical activity apps lack sufficient inclusion in health behavior change theories and evidence-based content [19]. However, using physical activity apps provides more convenience in terms of use and more flexibility regarding time. As such, physical activity apps have become increasingly popular [20].

According to a statistical report of the International Telecommunication Union [21], the total number of global internet users has increased from 1.99 billion in 2010 to 3.38 billion in 2016. By the end of 2017, the number of cellular mobile subscriptions totaled around 700 million, with a penetration rate of 70% even in the less developed countries. In addition, the scale of Chinese internet users totaled 802 million, of which mobile phone users accounted for 98.3%, as of June 2018 [22]. All types of scales for mobile app users are rising. In addition, users of mobile physical activity apps accounted for 78% of all users of mobile health–related apps in 2014, up from 39% in the previous year [23].

The influence of physical activity apps has been examined at the level of physical activity. Most existing studies have confirmed the promotional effect of physical activity apps on physical activity. One study of undergraduates at the Southeast University, in Jiangsu Province, China, showed that only 27.85% of college students had never used physical activity apps [24], suggesting that physical activity apps may be extremely popular among Chinese college students. Another study [25] confirmed the role of physical activity apps in increasing the time spent on physical activity and suggested that such changes could further increase people’s self-efficacy. Harries [26] demonstrated the significant effect of physical activity apps on young people who lack physical activity but noted no significant increase in social feedback. However, other studies have highlighted that although physical activity apps may play a role in increasing physical activity, the mechanism is not clear and needs to be improved to achieve better results [27,28].

Social support and self-efficacy may be the factors that affect physical activity. Social support is defined as the exchange of resources between at least 2 individuals perceived by the provider or recipient to be intended to enhance the well-being of the recipient [29]. Self-efficacy is defined as the belief that one can successfully execute the behavior required to produce the desired outcomes [30]. Shariff [14] indicated that social support from family, friends, and coaches can influence the behavior of teenagers with regard to sports and psychological development and social competence. Zhang [15] reported that social support can play such a role with certain conditions. As such, the actual effect of social support on physical activity is not clear [31]. With regard to the effect of physical activity apps on self-efficacy, many results suggest that self-efficacy is a powerful predictor of physical activity. Bezjak [16] found a significant correlation between self-efficacy and involvement of college students in physical activity. Smith [32] showed that self-efficacy, as a mediating variable between physical activity apps and physical activity, affects its effect strength. This finding elaborates the specific mechanism of physical activity apps in improving physical activity levels.

In general, research on the influence of physical activity apps on physical activity level is incomplete. The results of existing works are contradictory, which may be attributed to the subjects and the region of study. Most studies have confirmed that the use of physical activity apps can promote physical activity levels. However, the degree of influence and mechanism of action remain unclear. As a special group, college students are highly adaptable and need to cultivate good physical activity habits. As such, college students represent an ideal research sample when investigating interventions.

Therefore, the study aimed to elucidate the mediating role of social support and self-efficacy in physical activity apps and physical activity, and on the specific mechanism of how use of physical activity apps is associated with physical activity level. Our findings will contribute to the development of physical activity interventions and improvement of health at the national level.

These are hypotheses of this study:

- The use of physical activity apps is associated with higher physical activity levels among college students.
- Physical activity apps may be associated with college students’ physical activity levels, mainly through the mediating effect of social support and self-efficacy.
- According to the conclusions of this study, a set of potential measures hypothesized to improve college students’ physical activity level with the help of physical activity apps can be formulated.

## Methods

### Participants

We conducted a closed survey. An electronic questionnaire was used to survey college students in Beijing from December 27, 2017, to January 5, 2018. Convenience sampling was adopted to issue the questionnaire. According to a previous survey, 43% of college students who use physical activity apps and 36% of those who had never used them achieved the WHO’s physical activity recommendations. Thus, the sample size was calculated to be 500. We contacted student union leaders and teachers at several colleges in Beijing and sent the electronic questionnaire through their WeChat (the Chinese version is Weixin) group. To obtain cooperation, an incentive (about 2 yuan per participant) was paid out with each questionnaire.

The questionnaire was distributed to a wide range of students through the WeChat group, not sent to individuals. Thus, it was difficult to calculate the exact number of questionnaires sent and the response rate. However, it was estimated that the response rate ranged from 70% to 95% in the different WeChat groups. Users first had to log in using their WeChat account to prevent multiple entries from the same individual. Only those who agreed to participate and completed the questionnaire could submit the completed questionnaire. Therefore, we could not calculate the exact view rate and participation rate.

We controlled the filling range of the data when designing the questionnaire to avoid the occurrence of invalid data. After collecting the electronic questionnaires, we manually checked the original data. Strict criteria were used to screen valid questionnaires. Only completed questionnaires with no missing data and no logical errors were considered valid. Otherwise, the questionnaire was excluded. For example, if the participant did not indicate a specific amount of time spent on physical activity, his/her questionnaire would be excluded. Finally, 1476 questionnaires were completed. After excluding invalid questionnaires, 1245 valid questionnaires were used in this analysis, with an efficiency rate of 84.35%.

### Measures

The questionnaire consisted of 5 parts: use of physical activity apps, sports habits, social support, self-efficacy, and social demographic information. The electronic questionnaire consisted of 5 pages, and each page included 15 items. We used adaptive questioning (only conditionally displayed based on responses to other items) to reduce the number and complexity of questions. In addition, respondents were able to review and change their answers before submitting the questionnaire. Before fielding the questionnaire, we asked experts for their advice and tested the usability and technical functionality of the electronic questionnaire on a small scale.

In the research, those who used physical activity apps in the past month were defined as current users, those who had used physical activity apps before but not in the past month were defined as past users, and those who had never used physical activity apps were defined as nonusers.

The measurement of physical activity habits was based on the Chinese simplified IPAQ [33], which is mainly used to evaluate people’s physical activity level against the recommended level. It can also be used to evaluate the results of a physical activity intervention. In 2004, Chinese scholars Qu and Li studied the reliability and validity of the Chinese version of IPAQ. Their results indicated that the retest reliability and validity are higher than or equal to the questionnaire for the same use [34]. Thus, the Chinese version of the questionnaire was used in the present research.

To measure social support, we consulted Chogahara’s research on older adults [33] and Cavallo’s study on college girls [35]. Social support was delineated as partnership, information, and respect support types. Then, 2 subevaluation indicators were selected from each aspect, which respondents graded according to the actual frequency of occurrence on a scale ranging from 1 (never) to 5 (often). In addition, a homogeneity reliability test and factor analysis were performed. The results of the sphericity test confirmed that Cronbach alpha was .917, KMO (Kaiser-Meyer-Olkin)=0.887, and the *P* value was ＜.001. The reliability and validity of this scale were considered good.

The evaluation of self-efficacy was based on Wang’s study on adolescents with disabilities [36] and Ashraf’s research on adolescent girls [37]. A 5-point Likert scale with 6 items was used. The participants rated every item of the scale, according to their confidence level regarding participating in physical activity in different situations. Each item was scored on a scale ranging from 1 (very uncertain) to 5 (very certain). In addition, a homogeneity reliability test and factor analysis were performed. The results of the sphericity test showed that the Cronbach alpha was .914, KMO=0.906, and the *P* value was ＜.001. Again, the reliability and validity of the scale were considered good.

### Ethics Statement

This study has been approved by the Peking University Institutional Review Board Office. Before participating in the study, each participant was informed of the purpose of the investigation and the duration of the survey and assured that the results would be used only for the purpose of this study, and that their privacy would be guaranteed. If participants did not want to participant in the survey, they could simply turn off the electronic questionnaire and drop out. If the questionnaire was completed and submitted, the participant was considered to have provided informed consent. Only those who voluntarily agreed to participate in the survey were included in the research.

### Statistical Analyses

Data were analyzed using SPSS (IBM) version 22.0 and Mplus (Linda Muthén & Bengt Muthén) version 7.0. A descriptive analysis was conducted to analyze the social demographic information and use of physical activity apps. A chi-square test was carried out on the difference in rates between the groups. The Kruskal-Wallis rank test and Mann-Whitney *U* test were performed for the differences in ordinal level variables between groups. The correlation between variables was tested linearly. Structural equation modeling (SEM) was used to test the relationships between the use of physical activity apps, self-efficacy, social support, and level of physical activity. A *P* value <.05 was considered statistically significant. SEMs were employed for the mediation effect analysis.

First, we indicated self-efficacy as a mediator between physical activity app usage (app use) and physical activity to test whether physical activity app usage has a significant indirect effect on physical activity through self-efficacy and whether this indirect effect can completely explain the relation between app use and physical activity. App use was clarified as a 3-level categorical predictor (current users=3, past users=2, and nonusers=1), with self-efficacy as the mediator and physical activity as the outcome variable. The 6-item self-efficacy scale indicators were categorized in 3 parts (bad mood, support deficiency, and time deficiency), representing the manifest indicators by which self-efficacy was significantly explained (*P*<.001). The durations of VPA and MPA were also set as manifest indicators by which physical activity can also be significantly explained.

Social support was selected as the second mediator in the mediation model. The 5-item social support scale indicators were classified in 3 parts (partnership, information, and respect support), representing the manifest indicators by which social support was significantly explained (*P*<.001).

Finally, we added age and gender as covariables to the model. Gender was a 2-level categorical variable, where male=1 and female=2, whereas age was a continuous variable. The model was set to test for whether the direct and indirect effects of app use on physical activity changed with the combination of these covariables and how gender and age affected app use.

## Results

### Demographic Information

Of the 1245 participants, 466 were male (37.4%). Other demographic characteristics are shown in Table 1.

### Usage of Physical Activity Apps

**Figure 1:**
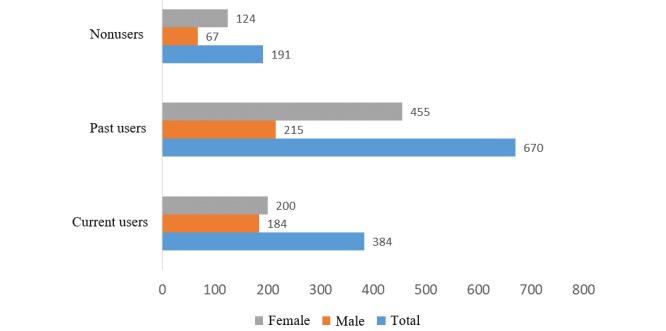
Number of each type of app user (male and female).

There was a difference between males and females in the distribution of the 3 types of users (χ^2^=26.6, *df*=2, *P*<.001). Other information are shown in Figure 1.

Of 384 current users, 99 (25.8%) indicated having used physical activity apps more than 4 times a week, 39.1% (150/384) used them 2 to 3 times a week, and 35.1% (135/384) used apps less than once a week. The popular functions current users mostly used were *calculating the number of steps* (54.9%, 211/384), *recording movement* (52.9%, 203/384), the *training plan* (49.2%, 189/384), *recording calorie consumption* (40.6%, 156/384), and the *video coach* (40.6%, 156/384). Functions such as *ranking campaign* (20.8%, 80/384), *sharing exercise data on social media* (11.7%, 45/384), and *stimulating users* (7.3%, 28/384) were less used by the current users.

**Table 1:** Participants’ demographic information.

Demographics		Total (N=1245)	Male (n=466)	Female (n=779)	Chi-square *t* test (*df*)^a^	*P* value^b^
Age (years), mean (SD)		20.5 (2.6)	20.8 (2.7)	20.3 (2.4)	2.99 (1244)	.003
Body mass index (kg/m^2^), mean (SD)		22.3 (6.5)	23.6 (7.0)	21.5 (5.8)	5.55 (1244)	<.001
**Grade, n (%)**					13.0 (4)	.01
	One	190 (15.3)	52 (11.2)	138 (17.7)		
	Two	357 (28.7)	135 (29.0)	222 (28.5)		
	Three	295 (23.7)	127 (27.3)	168 (21.6)		
	Four and five	286 (23.0)	104 (22.2)	182 (23.3)		
	Graduate student or above	117 (9.4)	48 (10.3)	69 (8.9)		
**Birthplace, n (%)**					6.6 (2)	.04
	Urban area	858 (68.9)	305 (65.5)	533 (71.0)		
	Rural area	360 (28.9)	146 (31.3)	214 (27.5)		
	International students	27 (2.2)	15 (3.2)	12 (1.5)		
**Ethnicity, n (%)**					3.7 (1)	.06
	Han	1079 (86.7)	415 (89.1)	664 (85.2)		
	Others	166 (13.3)	51 (10.9)	115 (14.8)		
**Average monthly expense (yuan), n (%)**					6.1 (4)	.19
	<500	36 (2.9)	18 (3.9)	18 (2.3)		
	500 to 1000	191 (15.3)	80 (17.2)	111 (14.2)		
	1000 to 1500	374 (30.0)	139 (29.8)	235 (30.2)		
	1500 to 2000	319 (25.6)	107 (23.0)	212 (27.2)		
	>2000	325 (26.1)	122 (26.2)	203 (26.1)		

^a^The age and the body mass index was tested using a *t* test. Others were tested using a Pearson chi-square test.

^b^The *P* value here refers to whether the difference in these demographic characteristics between participants of different genders was statistically significant.

### Physical Activity

Normality tests were conducted for concerning variables, and the results of these did not support the null hypothesis. Thus, we used quartiles to describe the distribution. The median duration of VPA per week was 30 min and 40 min for MPA. Overall, only 48.3% (602/1245) of the participants attained the WHO’s weekly standard amount of physical activity.

For all types of physical activity, we noted a significant difference between groups (Table 2): Current users reported the most VPA and MPA time consumption, and nonusers reported the least time spent on these physical activity types. For social support, current users had the highest score (median=21), followed by past users (median=18). Nonusers scored the lowest (median=17; *P*<.001). There was also a significant difference in the self-efficacy scores of these groups (*P*<.001).

The physical activity rates of participants who attained the WHO’s recommended value in the 3 groups were calculated. A cross-table test was performed, revealing a significant difference between the 3 physical activity apps usage groups (χ^2^=385.0; *P*<.001). Current users had the highest rate (66.75%) and nonusers had the lowest (35.94%; Table 2).

### Relationship Between Physical Activity App Usage, Physical Activity, Social Support, and Self-Efficacy

The zero-order correlations between measures are displayed in Table 3. Current users scored higher for social support and self-efficacy and reported more physical activity. Gender (male=1, female=0) was significantly correlated with all measurements, demonstrating that this factor might be an assignable confounding variable. Self-efficacy was significantly correlated with all measurements, demonstrating that it could be an intensive mediator.

Age was also significantly correlated with all measurements, except physical activity app usage, revealing that it could also be a confounding variable. BMI was significantly correlated with gender, age, and self-efficacy, but not with social support and physical activity. Social support was significantly correlated with all measurements, except BMI, and the same was observed for physical activity. Overall, BMI may not be an important factor in our research.

**Table 2:** Differences between current users, past users, and nonusers.

Items per week		Current users (n=384)			Past users (n=670)			Nonusers (n=191)			Kruskal-Wallis and Mann-Whitney *U* rank sum tests, chi-square (*df*)^b^	*P* value
		P25^a^	M^a^	P75^a^	P25^a^	M^a^	P75^a^	P25^a^	M^a^	P75^a^		
**Time, VPA^c^** **(min)**												
	Total	23	60	126	0	30	80	0	2	73	104.36 (1242)	<.001
	Male	17	60	120	2	30	90	0	20	90	43.87 (463)	<.001
	Female	37	90	180	0	25	60	0	0	60	49.42 (776)	<.001
**Time, MPA^d^** **(min)**												
	Total	20	60	150	1	30	84	0	20	90	59.11 (1242)	<.001
	Male	20	60	120	2	40	100	0	30	120	26.31 (463)	<.001
	Female	22	90	180	0	30	80	0	20	60	27.45 (776)	<.001
**Walk time (min)**												
	Total	50	140	280	45	120	210	21	105	210	9.42 (1242)	.009
	Male	60	140	280	50	125	250	40	140	315	0.27 (463)	.88
	Female	45	140	247	45	120	210	11	90	120	14.60 (776)	.88
**Sedentary behavior (min)**												
	Total	60	600	1698	120	840	2100	50	630	2100	6.53 (1242)	.04
	Male	60	420	1440	60	720	2100	60	630	2100	0.46 (463)	.80
	Female	90	760	2100	180	900	2100	42	647	1890	14.34 (776)	.80
**Social support score**												
	Total	14	21	25	12	18	22	11	17	21	156.67 (1242)	<.001
	Male	14	19	24	12	18	22	11	17	21	21.87 (463)	<.001
	Female	14	21	25	12	18	22	12	16	19	20.03 (776)	<.001
**Self-efficacy score**												
	Total	16	20	24	12	16	19	10	16	19	124.66 (1242)	<.001
	Male	16	19	23	12	17	21	11	17	21	38.72 (463)	<.001
	Female	17	21	25	12	16	18	9	15	18	75.93 (776)	<.001
**Rate of reaching the WHO^e^ standard (%)**												
	Total	66.7			42.1			35.9			385.0 (1242)	<.001
	Male	77.4			49.8			46.3			23.3 (463)	<.001
	Female	60			38.5			29.8			19.5 (776)	<.001

^a^P25 is the upper quartile, M is the median, and P75 is the lower quartile.

^b^The rate of reaching the WHO standard was tested using a Pearson chi-square test. Others were tested using Kruskal-Wallis and Mann-Whitney *U* rank sum tests.

^c^VPA: vigorous-intensity physical activity.

^d^MPA: moderate-intensity physical activity.

^e^WHO: World Health Organization.

**Table 3:** Zero-order correlations between measures.

Measures^a^	(1)	(2)	(3)	(4)	(5)	(6)	(7)
App use (1)	1.000	—^b^	—	—	—	—	—
Gender (2)^c^	–.112^d^	1.000	—	—	—	—	—
Age (3)	.050	.084^d^	1.000	—	—	—	—
Bpdy mass index (4)	–.006	.156^d^	–.230^d^	1.000	—	—	—
Social support (5)	.375^d^	.133^d^	–.130^d^	–.047	1.000	—	—
Self-efficacy (6)	.298^d^	.158^d^	–.119^d^	–.066^d^	–.808^d^	1.000	—
Physical activity (7)	.111^d^	.142^d^	–.060^d^	–.031	–.288^d^	–.273^d^	1.000

^a^Numbers in parentheses correspond to column numbers.

^b^To avoid duplication of data and to keep tables concise, there are empty cells in the table.

^c^Gender: 1=male, 0=female.

^d^Correlation is significant at the .001 level (2-tailed).

### Simple Mediation Model

**Figure 2:**
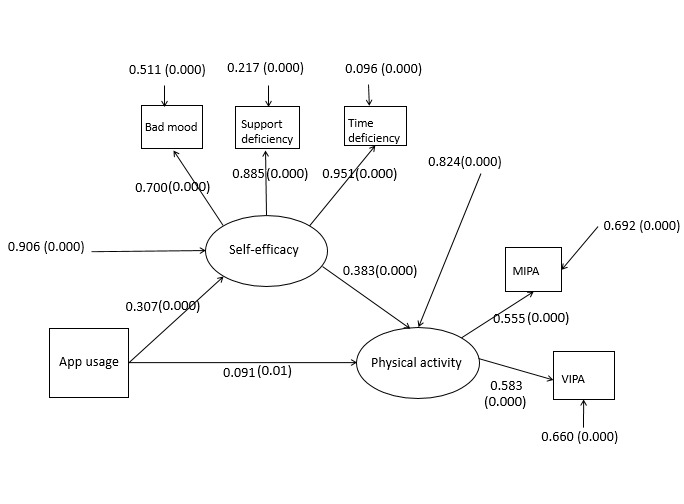
Single mediation model, in which app-usage is a three-level categorical predictor (current users=3, past users=2, and non-users=1). Self-efficacy is described by bad mood, support deficiency, and time deficiency; VIPA is vigorous-intensity physical activity and MIPA is moderate-intensity physical activity. (0.000) represents significant path coefficients at the .001 level and (0.01), at the .05 level.

The standardized path coefficients of the simple mediation model are displayed in Figure 2. The model fit indices were as follows: χ^2^=2.9, comparative fit index (CFI)=.995, standardized root mean square residual (SRMR)=.018, and root mean square error of approximation (RMSEA)=.039. The single mediation model indicated that the path coefficient of the indirect effect was .118, slightly higher than the coefficient of the direct effect (.091). Furthermore, it showed that the use of an app was weakly associated with a higher physical activity level and strongly associated with a higher physical activity level through self-efficacy.

### Multi-Serial Mediator Model

**Figure 3:**
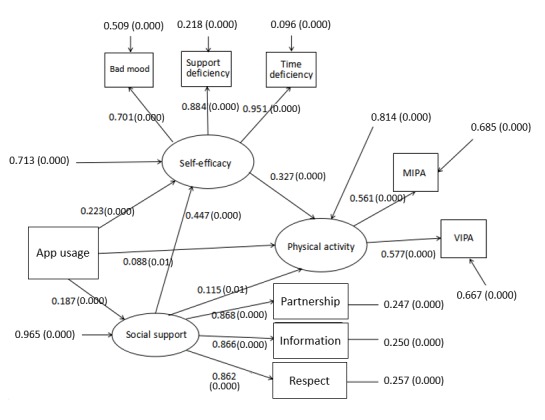
Multi serial mediator model, in which social support is described by three manifest indicators (partnership support, information support, and respect support). Self-efficacy is described by bad mood, support deficiency, and time deficiency; VIPA is vigorous-intensity physical activity and MIPA is moderate-intensity physical activity. (0.000) represents significant path coefficients at the .001 level and (0.01), at the .05 level.

Self-efficacy was affected by social support directly, meaning that social support had a second role in this model, namely as a serial mediator between app use and self-efficacy, according to self-efficacy theory. Furthermore, social support also acted as a sole mediator between app use and physical activity. The standardized path coefficients are displayed in Figure 3. The model fit indices were as follows: χ^2^=3.4, CFI=.990, SRMR=.018, RMSEA=.044. The multi-serial mediator model demonstrated that the use of an app was associated with higher physical activity through social support. In addition, the combination of social support in this model did not change the significant indirect effect of self-efficacy and direct effect of app use on physical activity. Moreover, the association between app use and higher physical activity through self-efficacy and social support was stronger than the association directly between app use and higher physical activity. Compared with the single mediation model, (1) the combination of social support did not change the lower direct effect compared with the total indirect effect; (2) the association between app use and higher physical activity level and between app use and higher physical activity level through self-efficacy weakened with the combination of social support and (3) the association between app use and higher physical activity level through social support was weaker than the association between app use and higher physical activity level through self-efficacy.

### Multi-Serial Mediator Model Containing Covariables

**Figure 4:**
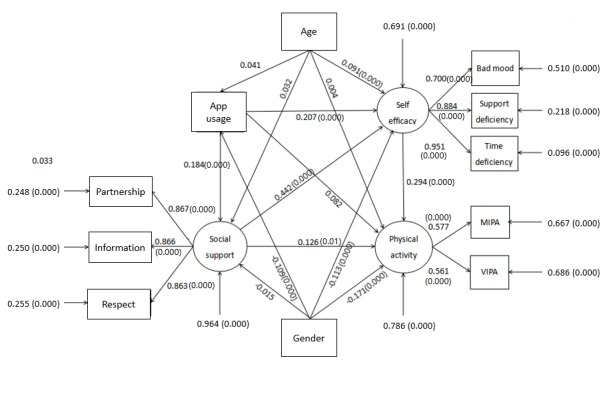
Multi serial mediation model containing age and sex. Age is a continuous co-variable; gender is described as a two-level categorical variable where male=1 and female=2. Social support is described by three manifest indicators (partnership support, information support, and respect support). Self-efficacy is described by bad mood, support deficiency, and time deficiency; VIPA is vigorous-intensity physical activity and MIPA is moderate-intensity physical activity. (0.000) represents significant path coefficients at the .001 level and (0.01), at the .05 level.

The standardized path coefficients of the multi-serial mediator model containing covariables are displayed in Figure 4.

The model fit indices were as follows: χ^2^=3.1, CFI=0.987, SRMR=0.018, RMSEA=0.041. The model showed that the path coefficients from gender to app use, self-efficacy, and physical activity were all significant at the .001 level, whereas the path coefficient was only significant from age to self-efficacy (*P*<.001). For gender, males had a higher app use rate and higher social support and self-efficacy scores. Furthermore, they reported more physical activity. Regarding age, older students tended to obtain higher scores for self-efficacy. Adding age and gender into this model did not change the association between app use and higher physical activity level through self-efficacy and social support. However, the model indicated that there was no longer a direct association between app use and higher physical activity level after including the covariant items. The standardized direct effect calculated in Mplus7.0 was 0.082 (*P*=.05) slightly lower than the total indirect effect of social support (0.084; *P*<.001), of which self-efficacy was 0.061 (*P*<.001) higher than social support (0.023; *P*<.05). Compared with the multi-serial mediation model, this model provided the following information: (1) the combination of age and gender did not change the situation of the weaker association directly between app use and higher physical activity, compared with the association through self-efficacy and social support; (2) with the combination of age and gender, the association through self-efficacy and social support became weaker; (3) the association through social support was still weaker than the association through self-efficacy.

## Discussion

### Principal Findings and Interpretation

This study confirmed the correlation between the use of physical activity apps and the level of physical activity. The use of physical activity apps is associated with a higher social support level and higher self-efficacy score. Furthermore, a high social support level and high self-efficacy score are associated with higher physical activity levels.

The results showed that 13.3% of college students had never used physical activity apps, less than the percentage of students who never used physical activity apps which found in a survey of college students in Jinan [38]. However, the difference may be attributed to regional and methodological differences. Furthermore, this research confirmed the association between use of physical activity apps and physical activity levels, coinciding with a previous research on undergraduates in Southeast University [23].

Research in the United States [39] has shown that 55.4% of adults aged 18 to 24 years met the aerobic guidelines of the US Department of Health and Human Services, whereas 26.9% were totally inactive. In Beijing, 27.3% of students exercised regularly (3 times a week for 30 minutes each time) [40]. In this study, 66.75% of college students who were using physical activity apps met the WHO’s recommended level. This could be attributed to both population differences and the different methodologies used to measure activity. However, it can still be concluded that college students who were using physical activity apps had a higher level of physical activity. Among college students who had never used physical activity apps, 35.94% met the WHO’s recommended level. This is perhaps because of school policies and physical education requirements, and perhaps because college students have received health education on physical activity from various sources. As far as we know, some colleges in Beijing require that students spend a certain amount of time on physical activity to earn credits. Therefore, particular proportion of college students in Beijing have a high level of physical activity. This can be further explored in future investigations. Thus, while the physical activity level of college students in Beijing is not low, but it still needs improvement.

Our findings showed that 49.7% of college students who used physical activity apps had app friends. App friends are network friends who follow each other on physical activity apps and engage in interactive behaviors such as *liking* a post. This kind of network friend can be friend in life, or not. According to the statistical analysis of the social support scores of different sources and classifications, social support from friends is related to VPA. This might be because friends on apps are mostly network strangers, and thus, the correlation is weaker. Social support requires specific conditions. The role of social support from networks only comes into play after a change in personal attitudes toward physical activity [15]. This tendency may also lead to more social support from actual friends than app friends. This study found that different types of social support had different effects on physical activity. Respect support is associated with VPA and total duration of physical activity, whereas partner support is associated with MPA. This may be attributed to different social support mechanisms, which can be further be explored and refined in subsequent studies.

Self-efficacy was significantly associated with physical activity, as revealed by earlier findings [41]. Among the app use groups, current users scored the highest for self-efficacy. Thus, app usage may strengthen college students’ self-efficacy in exercise, confirming earlier findings [27]. As previously reported, self-efficacy mediates the relationship between app use and exercise [25], indicating the effect of app use on self-efficacy. Furthermore, self-efficacy then influences physical exercise. Indeed, social support from family members, friends, and coaches is considered to affect adolescents’ physical activity–related behavior [14]. Self-efficacy was also associated with social support, indicating that self-efficacy may be predicted by social support to some extent. In a study on junior high school students in Shanghai, social support reportedly explained self-efficacy [42], which is consistent with our results. In the same study, social support and self-efficacy were employed in a mediation model, where self-efficacy mediated the effect of social support on satisfaction with youth physical activity. This study assumed social support as the first mediator of physical activity and mobile app usage, which can also affect self-efficacy.

The results of the multi-mediation model showed that the 2 mediators of social support and self-efficacy were both significant, with social support having a significant effect on self-efficacy. These results were consistent with those of previous studies that indicated the underlying mechanism between exercise app usage and physical activity. Moreover, for the indirect effect, self-efficacy had a greater impact (standardized coefficient: 0.073 of 0.094), indicating its contribution to the larger mediation effect compared with social support.

To control potential confounding variables, we selected age and gender as covariables in the mediation model. Our study appears to be the first to consider gender and age as covariables in a mediation model. The direct effect was no longer significant after controlling for these 2 variables in the model, indicating that the significant coefficient value between app use and physical activity may have been erroneous before covariables were added into the model. However, the controlled multi-mediation model confirmed the unchanged significant association of mediators (social support and self-efficacy) between app use and physical activity. This controlled mediation model also indicated that gender and age may be important covariables that need to be considered in future studies. This might be because gender is associated with self-efficacy and social support, and the older students become, the more they need to do, meaning they have less time for physical activity. Our research only focused on college students in Beijing, meaning that the age range was narrow. In future studies, more participants with a greater age range should be surveyed to obtain more convincing results.

This study had a number of limitations. First, given the characteristics of the electronic questionnaire, the samples were obtained through convenience sampling and the survey limited to several colleges in Beijing. At the same time, we could not obtain information on the differences in demographic characteristics of participants taking part and those who did not. As such, the representativeness of the survey participants may not be high. Second, the proportion of female students (62.6%) was higher than that of male students, with a notable statistical difference. This trend may be because the response rate for the questionnaire was higher for females. Third, we set strict data inclusion criteria, resulting in the slightly low efficiency of the questionnaire (84.35%). Finally, the survey was conducted in winter, which may have affected the level of physical activity reflected in the results. In future research, these limitations need to be addressed.

### Conclusions

This study focused on college students in Beijing and found that the use of physical activity apps is associated with higher physical activity levels among them. This effect is mainly through the mediation effect of social support and self-efficacy, rather than the direct effect of physical activity apps. The use of physical activity apps is associated with a higher social support level and the higher self-efficacy score. Finally, a high social support level and high self-efficacy score are associated with higher physical activity levels.

## Abbreviations

BMI: body mass index
CFI: comparative fit index
IPAQ: International Physical Activity Questionnaire
KMO: Kaiser-Meyer-Olkin
MPA/MIPA: moderate-intensity physical activity
PA: physical activity
SRMR: standardized root mean square residual
VPA/VIPA: vigorous-intensity physical activity
WHO: World Health Organization
